# A *cis*‐eQTL genetic variant in *PLK4* confers high risk of hepatocellular carcinoma

**DOI:** 10.1002/cam4.2487

**Published:** 2019-09-06

**Authors:** Lijuan Meng, Yan Zhou, Sihan Ju, Jing Han, Ci Song, Jing Kong, Yifei Wu, Shuai Lu, Jiani Xu, Wenwen Yuan, Erbao Zhang, Cheng Wang, Zhibin Hu, Yayun Gu, Rongcheng Luo, Xuehao Wang

**Affiliations:** ^1^ Integrated Hospital of Traditional Chinese Medicine Southern Medical University Guangzhou China; ^2^ State Key Laboratory of Reproductive Medicine Nanjing Medical University Nanjing China; ^3^ Department of Epidemiology School of Public Health Center for Global Health Nanjing Medical University Nanjing China; ^4^ Jiangsu Key Lab of Cancer Biomarkers Prevention and Treatment Collaborative Innovation Center for Cancer Personalized Medicine Nanjing Medical University Nanjing China; ^5^ Jiangsu Cancer Hospital & Jiangsu Institute of Cancer Research & The Affiliated Cancer Hospital of Nanjing Medical University Nanjing China; ^6^ Department of Bioinformatics School of Basic Medical Sciences Nanjing Medical University Nanjing China; ^7^ Department of Liver Surgery The First Affiliated Hospital with Nanjing Medical University Nanjing China

**Keywords:** aneuploidy, cancer‐testis gene, CFI‐400945, hepatocellular carcinoma, *PLK4*

## Abstract

**Purpose:**

The overexpression and knockdown of *PLK4* were both reported to generate aneuploidy. Thus, we aimed to investigate whether genetic variants in *PLK4* contribute to the development of hepatocellular carcinoma (HCC).

**Methods:**

We evaluated associations of common variants in *PLK4* and its promoter for the risk of HCC in our association study (1300 cases and 1344 controls). The genotype‐tissue expression (GTEx) and The cancer genome atlas (TCGA) databases were used to quantify the expression of *PLK4*. Cell proliferation and migration affected by PLK4 in HCC were assessed in vitro. Drug susceptibility testing (DST) model was used to assess the sensibility of *PLK4*‐activated HCC to CFI‐400945, a small molecule inhibitor of PLK4.

**Results:**

Herein, we found a significant association between rs3811741, located in the *PLK4* intron, and liver cancer risk (OR = 1.26, *P* = 9.81 × 10^−5^). Although *PLK4* expressed at lower levels in somatic tissues compared to the testis, the risk allele A of rs3811741 was associated with increased *PLK4* expression in liver cancer tissues. Additionally, *PLK4* high expression was remarkably associated with shortened survival of HCC (HR = 1.97, *P* = .001). Furthermore, overexpression of *PLK4* promoted, while knockdown of *PLK4* suppressed cancer cell proliferation, migration, and invasion. DST model demonstrated that CFI‐400945 can effectively suppress rampant proliferation of HCC with highly expressed *PLK4*.

**Conclusion:**

Taken together, our study demonstrated that *PLK4* is a susceptibility gene and plays an oncogenic role in HCC. Furthermore, we identified that *PLK4* sensitives HCC to CFI‐400945, which may be an ideal therapy target for HCC.

## Graphical abstract

*PLK4* is a newly defined cancer‐testis gene. It's *cis*‐eQTL genetic variant contributes to the development of hepatocellular carcinoma and sensitizes hepatocellular carcinoma (HCC) to CFI‐400945, which may be an ideal therapy target for HCC.

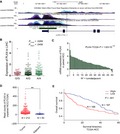

## 1 INTRODUCTION

Liver cancer is predicted to be the sixth most commonly diagnosed cancer and the fourth leading cause of cancer death worldwide in 2018, with about 841 000 new cases and 782 000 deaths annually.^1^ Hepatocellular carcinoma (HCC) is the most common type of liver cancer, accounting for approximately 90%.^2^ Major risk factors for HCC are chronic infections with the hepatitis B or C viruses (HBV/HCV), and exposure to dietary aflatoxin B1.^3^, ^4^ However, it is known that only a minority of HBV chronic carriers develop HCC, strongly suggesting the importance of genetic susceptibility for HBV‐related HCC.^5^ In the past decade, genome‐wide association studies (GWASs) have emerged as established tools in investigating common genetic variants associated with liver cancers.^6^ However, because of the stringent criteria of the GWAS significant level, the functional relevance of genetic variants, for the most part, has not been well characterized. Thus, it is still necessary to study the roles of functional candidates in the HCC susceptibility.^7^, ^8^

Cancer/testis (CT) genes belong to a family with predominant normal expression in testis, limited expression in other normal tissues and frequent expression in various types of cancers, reflecting certain similarities between gametogenesis and tumorigenesis.^9^, ^10^, ^11^ Polo‐like kinase‐4 (*PLK4*), a newly identified CT gene, is critical in regulating centriole duplication, which is required for mitosis and maintenance of chromosomal stability.^12^, ^13^, ^14^ In addition, *PLK4* overexpressed cancers shows mitotic fidelity defect, resulting in CIN and aneuploidy.^15^, ^16^ This shows that overexpression of *PLK4* results in aneuploidy and drives cancer development. Thus, we hypothesized that functional variants of *PLK4* might contribute to the development of HCC, especially those resulting in alterations in gene expression.

As a result, we found a functional *cis*‐expression quantitative trait loci (eQTL) genetic variant of the CT gene *PLK4*, rs3811741, associated with higher risk of HCC. In TCGA database, we discovered that *PLK4* was aberrantly activated and overexpressed in liver cancer tissues, in contrast with adjacent tissues. In addition, PLK4 played functional roles in supporting the tumorigenesis of HCC in vitro with multiple cells. Finally, we identified that PLK4 sensitives HCC to CFI‐400945, a small‐molecule inhibitor of PLK4 undergoing phase II clinical trial testing in breast cancer and prostate cancer,^17^, ^18^ which provided a potential therapy target for HCC.

## 2 MATERIALS AND METHODS

### 2.1 Subjects and variants selection

For the SNPs selection, we first extracted SNPs located in *PLK4* and its promoter regions (5 kb upstream of the transcription start site). We then conducted a quality control (QC) procedure, and SNPs with minor allele frequency (MAF) < 0.05, deviating from Hardy‐Weinberg equilibrium (HWE) in 1000 Genome (*P*_HWE_ < .05) and high linkage disequilibrium (LD)> 0.8, were excluded. The SNPs were scored for functions in 3DSNP (http://cbportal.org/3dsnp/). Finally, three functional SNPs were selected for genotyping by Sanger sequence. Patients were consecutively recruited between January 2006 and December 2010 at the Nantong Tumor Hospital (Nantong, China), Qidong Liver Cancer Institute (Qidong, China), and the First Affiliated Hospital of Nanjing Medical University without restrictions of age and sex, including 1300 HBV‐positive HCC patients and 1344 HBV‐positive controls.^5^ Association analyses were performed assuming an additive effect model adjusted for age, gender, and drinking status. Finally, we only selected the SNP with the lowest *P* value for further functional annotation and in vitro assays.

### 2.2 *Cis*‐eQTL analysis

The *cis*‐eQTL analysis was performed in liver hepatocellular carcinoma (LIHC) samples from TCGA database. First, to make the data better to the linear model, we transformed the gene expression levels across samples to the log 2 of RPKMs plus one. After, we performed a full linear regression analysis to detect the correlation between the genotype of rs3811741 and the expression of PLK4, with methylation and copy number alterations adjusted. The whole process was completed in R.^19^, ^20^

### 2.3 Cell culture

Human liver cancer cell lines (Huh7, BEL‐7402, MHCC‐97L, and MHCC‐97H) were obtained from the Cancer Research Institute of Southern Medical University, Guangzhou, China. Human liver cancer cell line G2.215 and immortalized human normal liver cell line L‐02 were kindly provided by School of Public Health, Nanjing Medical University, Nanjing, China. All cells were grown in Dulbecco's modified Eagle's medium (DMEM, Gibco, Invitrogen) with 10% fetal bovine serum (FBS, Biowest, France), and cultured in a humidified chamber with 5% CO_2_ at 37°C.

The source and mycoplasma contamination of the cell lines are, respectively, evaluated by Nanjing Cobioer Biosciences Co., Ltd and Beijing Microread Genetics Co., Ltd: DNA of the cell lines prepared using a DNeasy Blood & Tissue kit was analyzed by STR (Short tandem repeat) profiling. Our cell lines were considered to be identical to the ATCC or DSMZ corresponding cell lines when the entered STR profiles yield 100% match to the ATCC or DSMZ STR database. No cross‐contaminated cell lines or mycoplasma contamination was detected.

### 2.4 Transfection

Small interfering RNA (siRNA) specific for PLK4 was designed and synthesized by RiboBio Inc (Guangzhou, China) (sequence of siRNA is described in the Table S3). PLK4 plasmids were purchased in Genscript Technologies (Genscript, China). Six hours before transfection, Huh7 and BEL‐7402 cells were planted onto six‐well plates (Costar, America) at 75% confluence. siRNA and plasmids were then transfected at a working concentration using Lipofectamine 2000 (Invitrogen) or X‐tremeGENE HP DNA Transfection Reagent (Roche, Germany), respectively, according to manufacturer's protocols.

### 2.5 Immunoblotting

After the supernatant proteins were collected, concentrations were measured using BCA assay (Beyotime). Equal amounts of protein (40 μg) were then ran on a 10% sodium dodecyl sulfate‐polyacrylamide gel electrophoresis (SDS‐PAGE) gel. Gels were transferred to polyvinylidene fluoride (PVDF) membrane (Millipore), which was then incubated with specific primary antibody (Anti‐PLK4 antibody, ab 137398, Abcam). Subsequently, anti‐rabbit HRP‐conjugated secondary antibody (Beyotime) was applied, followed by the detection with the ECL solution (Millipore) and gel imaging system (Bio‐Rad).

### 2.6 Quantitative real time PCR assay

Total RNA was extracted by Trizol (Takara Bio, Inc.), and cDNA was synthesized using a reverse transcription kit (Vazyme). Quantitative real time PCR was carried out in triplicate with Power SYBR Green Master Mix on an Applied Biosystems QuantStudio 7 Flex Real Time PCR machine. The primer sequences for PLK4 were: forward 5′‐ GGACT GCGTG AAGGA AGCTA‐3′ and reverse 5′‐TGCAA CTTCC AAACC AGTGT G‐3′. Specificity of amplification products was confirmed by melting curve analysis. Results were normalized to the housekeeping gene GAPDH.

### 2.7 Cell viability analysis

The rate of in vitro cell proliferation was assessed using 3‐ (4,5‐dimethylthiazol‐2‐yl)‐2, 5‐diphenyltetrazolium bromide (MTT) assay. Cells were seeded in 96‐well plates at an appropriate density (Huh7, 3500‐4000 cells/well), and the absorbance value (OD) of formazan crystals was measured at 490 nm. Five wells were used, and experiments were performed three times. Clonogenicity assay was performed using six‐well dishes (Huh7, 1000 cells/well), and each cell group had six wells. The number of colonies containing more than 50 cells was counted. We employed transwell chambers (8‐umol/L pore size; Costar) with the bottom surface covered by fibronectin or matrigel to assay the invasion and migration ability. Cells adhering to the lower surface were fixed and then stained by crystal violet. Five fields per insert were randomly chosen to be counted by two independent investigators. All assays were independently repeated at least thrice. To examine cells’ migration ability, we also performed scratch wound‐healing assay. Primarily, transiently *PLK4* knockdown or overexpressing Huh7 and BEL‐7402 cells were seeded in 6‐well plates at 5 × 10^5^/well, and a single scratch was made. Images were captured under a microscope at 24, 48, 72, and 96 hours post wounding. Three independent experiments were performed in triplicate.

### 2.8 Drug sensitivity assay

The sensitivity to PLK4 inhibitor of HCC cells with different *PLK4* expression level was evaluated. CFI‐400945 (Selleck, USA), PLK4 specific inhibitor, was dissolved in DMSO to form a 10‐mM stock solution and stored at –80°C. Cells were seeded in 96‐well plates in 100ul DMEM medium supplemented with 10% FBS at a proper density (Huh7, 3500‐4000 cells/well). After incubation for 24 hours, supernatant was discarded and cells were continued to culture for 5 days with CFI‐400945 gradient concentration medium (0.1, 1, 10, 100, 1000 nmol/L). Drug sensitivity was determined by MTT assay. Experiments were carried out three times. Cell viability was calculated as follows: Viability (%) = [experimental group (OD) – blank group (OD)] / [control group (OD) – blank group (OD)] × 100.

### 2.9 Statistical analysis

We performed different expression analysis in 50 paired HCC tissues of TCGA database. We employed Cox analyses adjusted for age, gender, and tumor stage in the HCC samples of TCGA database. The data in this study were presented as means ± standard deviation (SD) from at least three independent experiments. Differences between the experimental and control groups were assessed by Student's *t* test or one‐way analysis of variance (ANOVA). A *P*‐value of .05 or less was considered statistically significant. Statistical analyses were computed using GraphPad Prism 6.0.

## 3 RESULTS

### 3.1 A *cis*‐eQTL SNP in PLK4 contributes to the development of HCC

**Figure 1:**
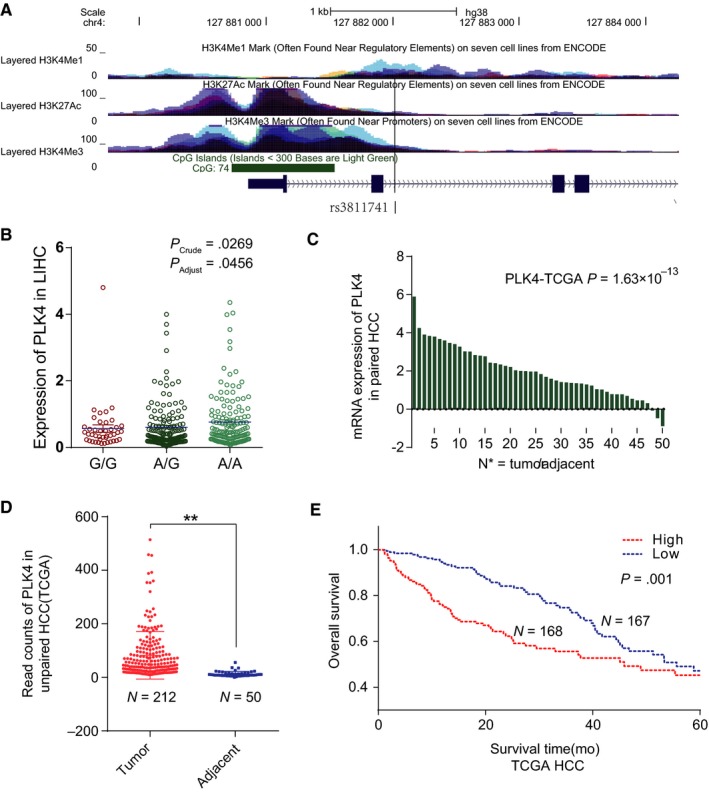
*PLK4* is reactivated in hepatocellular carcinoma (HCC) and is related with poor survival. A, Rs3811741 is located on the enhancer of *PLK4* modified by H3K4Me1 and H3K27Ac. B, *Cis*‐eQTL analysis of rs3811741 in tumor tissues of HCC based on data from the TCGA project, *P* value before and after the adjustment for methylation and copy number alterations were calculated. C, 50 paired HCC transcriptional data in TCGA was surveyed. *P*_paired_ *_t_*_ test_ < .01; N_paired_ = 50. D, Unpaired transcriptional data in TCGA were surveyed as well to show the differential expression of *PLK4* in tumor and adjacent tissues. Data are expressed as the mean ± SEM. **P_t__ test_ < .01; N_tumor_ = 346, N_adjacent_ = 60. E, Kaplan‐Meier curve of overall survival using patients’ data in TCGA. N_TCGA_ = 346. The cutoff we used to classify “high” and “low” *PLK4* expression was auto‐selected

To search for functional variants of *PLK4* contributing to the risk of HCC, we first selected SNPs within the gene body and the promoter region of *PLK4* in our previous HCC GWAS data and 19 SNPs were included in the initial analysis. These SNPs were then scored for functions in 3DSNP (http://cbportal.org/3dsnp/) (Table S1). Finally, three functional SNPs were selected for genotyping and association analysis. The genotyping was conducted by Sanger sequence. The primer sequences are summarized in Table S2. Association analysis was performed after assuming an additive effect model adjusted for age, gender and drinking status. As a result, functional SNP rs3811741 in *PLK4* showed the most significant association with HCC in the association study (rs3811741, OR = 1.26, *P* = 9.81 × 10^−5^, Table 1). Rs3811741 is located on the enhancer of *PLK4*, which is strongly modified by histone H3K4Me1 and H3K27Ac (Figure 1A), and might results in aberrant transcription of *PLK4*. Considering the limited expression of PLK4 in normal liver tissues except for testis, we performed the eQTL analysis in TCGA database rather than GTEx. And we found that rs3811741 presented *cis*‐eQTL with *PLK4* in HCC tumor tissues after adjustment for the CpG methylation level and copy number alterations (Figure 1B).

**Table 1:** Association between the SNPs and HCC susceptibility

Rs ID	Gene	Reference/Effect allele	Cases (1300)^a^	Controls (1344)^a^	MAF cases	MAF controls	OR (95% CI)^b^	*P* b
rs3811741	*PLK4*	G/A	606/558/136	525/629/190	0.32	0.38	1.26 (1.12‐1.41)	9.81 × 10^−5^
rs138343126	*PLK4*	G/T	81/510/709	88/539/717	0.26	0.27	0.96 (0.85‐1.09)	5.27 × 10^−1^
rs77248006	*PLK4*	G/A	16/260/1024	11/235/1098	0.11	0.10	1.18 (0.99‐1.41)	6.91 × 10^−2^

Abbreviations: HCC, hepatocellular carcinoma; MAF, minor allele frequency.

a Major homozygote/heterozygote/rare homozygote between case and control subjects.

b OR (95% CI) and *P *are derived from logistic regression analysis afer adjusting for age, sex, drinking, and smoking status.

We further analyzed the expression pattern of *PLK4* based on the GTEx database, and found that *PLK4* was highly and restrictedly expressed in the testis while extremely low in other tissues (Figure S1A). Above result was further confirmed by RT‐PCR assays in 16 normal tissues (Figure S1B). Moreover, the *PLK4* mRNA expression was aberrantly activated in HCC tumor tissues compared with adjacent tissues both in paired and unpaired tissues in TCGA database (*P* < .001; Figure 1C,D). In addition, Kaplan‐Meier analysis revealed that high *PLK4* expression was significantly associated with shorter overall survival of HCC (HR = 2.03, *P* = 1.2 × 10^−3^; Figure 1E). These results suggested that risk allele A of SNP rs3811741 might promote HCC development through the positive regulation of *PLK4* transcription.

### 3.2 Knockdown of PLK4 inhibits vitality of HCC cells

**Figure 2:**
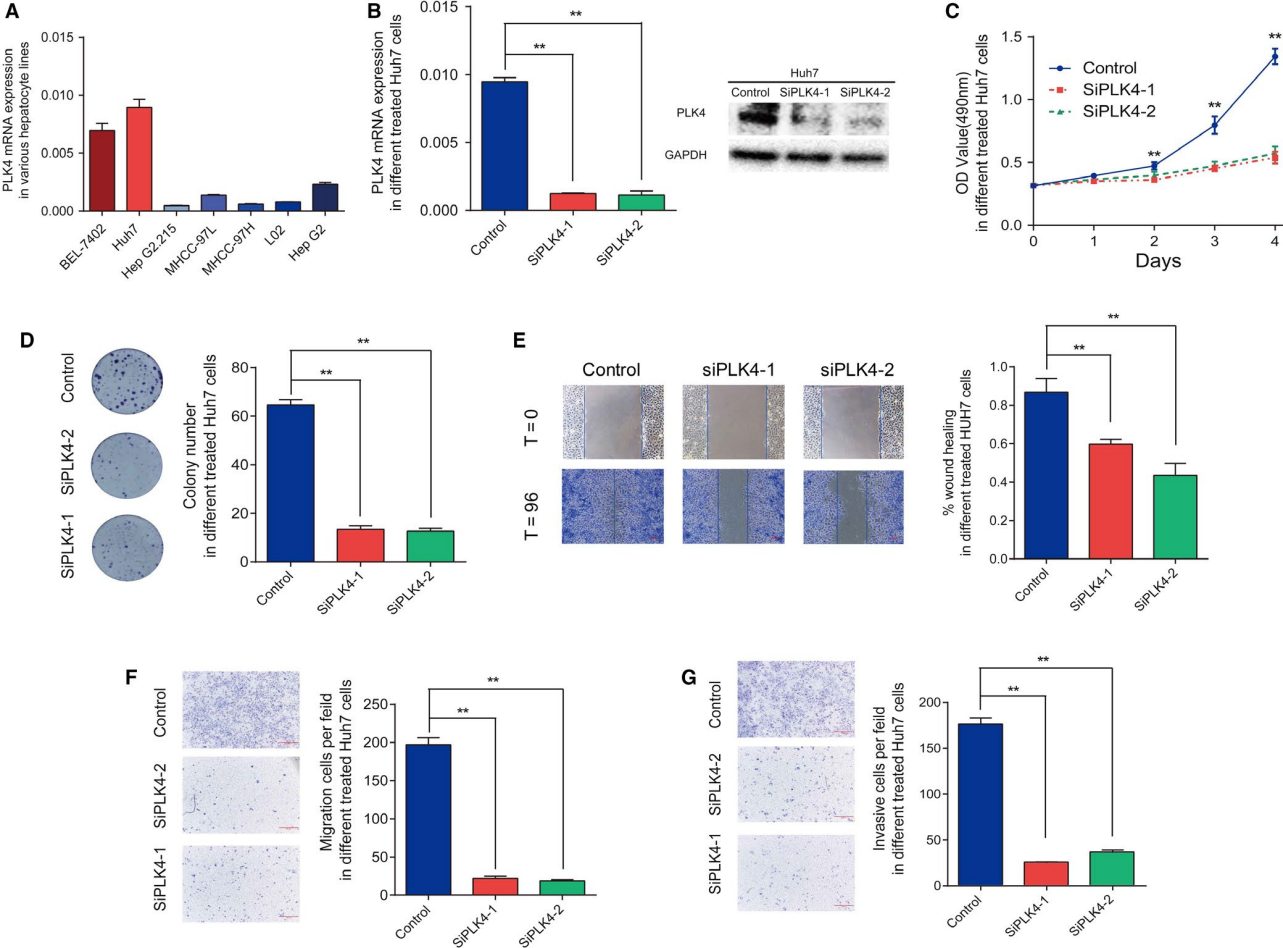
PLK4 is critical for HCC cell proliferation and migration. A, mRNA expression of PLK4 in different HCC cell lines. B, mRNA and protein expression of PLK4 were knockdown in Huh7 cells. C, MTT assays were used to determine the viability after knocking down *PLK4* in Huh7 cells. Data are expressed as the mean ± SEM. **P < .01. D, Clonogenicity assay in *PLK4*‐downregulated Huh7 cells showed the clonal formation ability. Data are expressed as the mean ± SEM. **P < .01. E, Scratch wound‐healing assay was performed to determine the migration of PLK4 knockdown Huh7 cells. Data are expressed as the mean ± SEM. **P < .01. F‐G, Cell migration and invasion ability were assessed by Transwell chambers in *PLK4*‐downregulated Huh7 cells. Data are expressed as the mean ± SEM. **P < .01

To investigate the biological role of PLK4 in HCC, we first investigated the expression of *PLK4* in six HCC cell lines (Huh7, BEL‐7402, G2.215, MHCC‐97L, MHCC‐97H, and HepG2) and one immortalized human normal liver cell line L‐02, and found that *PLK4* was highly expressed in HCC cell lines, especially Huh7, compared to human normal liver cell line both in mRNA and protein levels (Figures 2A and 4A). So, we knocked down *PLK4* by two siRNAs in Huh7. The efficiency of silence was validated at the mRNA and protein levels (Figure 2B). MTT assay and colony formation assay showed that the cell proliferation rate and colony formation ability were significantly reduced relative to controls (Figure 2C,D). Wound‐healing assay showed decreased migration abilities in *PLK4* knockdown cells (Figure 2E). Transwell assays also show that knockdown of *PLK4* resulted in decreased cell migration and invasion (Figure 2F,G).

### 3.3 PLK4 overexpression promotes cell viability of HCC cells

**Figure 3:**
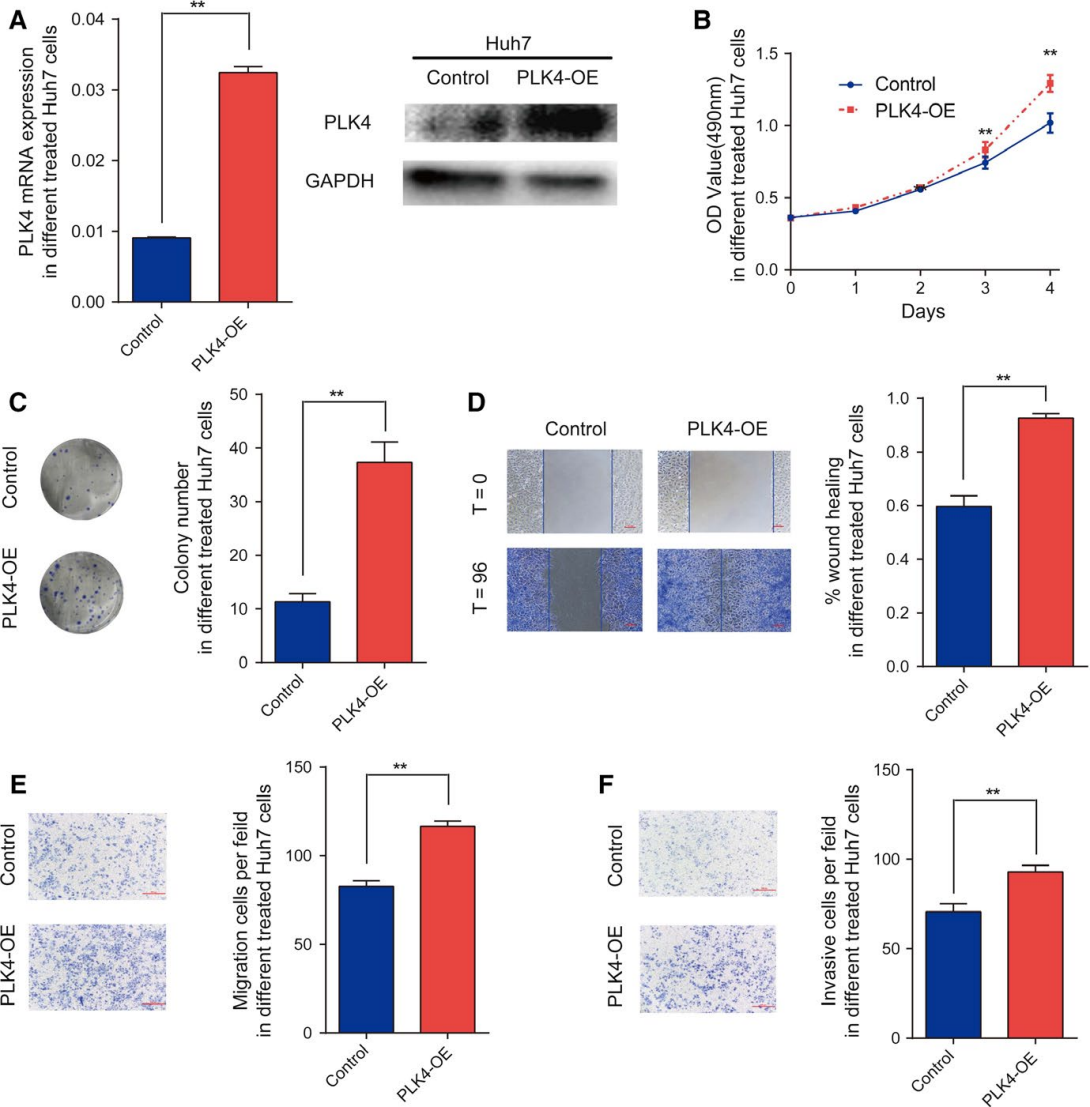
Overexpression of *PLK4* promotes HCC cell proliferation and migration. A, The mRNA and protein expression of *PLK4* was significantly higher in *PLK4*‐overexpressed Huh7 cell lines. B, MTT assays were used to determine the viability after *PLK4* overexpression in Huh7 cells. Data are expressed as the mean ± SEM. *P < .01. C, Clonogenicity assay in *PLK4*‐overexpressed Huh7 cells showed the clonal formation ability. Data are expressed as the mean ± SEM. **P < .01. D, The migration ability of *PLK4*‐ overexpressed Huh7 cells was determined by scratch wound‐healing assay and cell migration assay. Data are expressed as the mean ± SEM. **P < .01. E‐F, Cell migration and invasion ability were assessed by Transwell chambers in *PLK4*‐ overexpressed Huh7 cells. Data are expressed as the mean ± SEM. **P < .01

Meanwhile, we overexpressed *PLK4* with plasmids in Huh7 and the overexpression efficiency was verified at the mRNA and protein level (Figure 3A). As a result, MTT assay and colony formation assay revealed that *PLK4* overexpression increased cell growth rate (Figure 3B,C). Wound‐healing assay and transwell assay showed significantly increased migration abilities as compared to control (Figure 3D). Transwell assay also showed that overexpression of *PLK4* resulted in increased cell invasion (Figure 3E,F). Taken together, we conclude that *PLK4* may act as an oncogene in HCC tumorigenesis.

### 3.4 PLK4 sensitives HCC to CFI‐400945 inhibits

**Figure 4:**
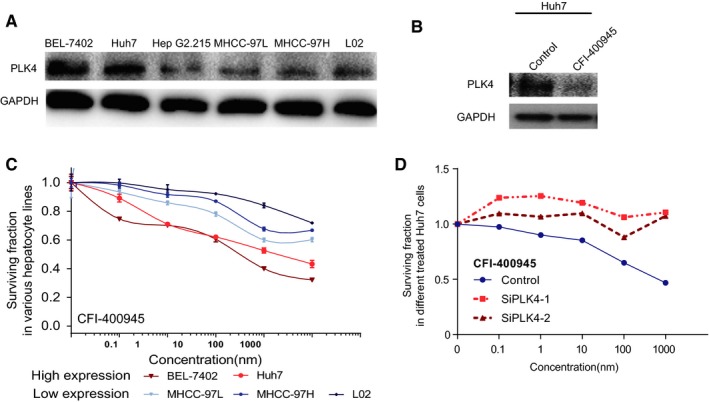
PLK4 increases sensitivity of HCC to PLK4 inhibitors. A, The protein expression of *PLK4* was significantly higher in Huh7 and BEL‐7402 cell lines. B, The protein expression of *PLK4* was inhibited in CFI‐400945 treated cell line. C, Drug sensitivity assay of CFI‐400945 was performed in various cell lines with both high and low background *PLK4* expression. The efficiency was evaluated using MTT assay. D, *PLK4*‐downregulated Huh7 cells treated with gradient concentrations of CFI‐400945, and the inhibition effect was assayed by MTT

To explore the prospect of clinical application, we conducted DST of CFI‐400945, a small molecule inhibitor of PLK4 undergoing phase I/II clinical trial testing (NCT03187288; NCT01954316; NCT03624543; NCT03385655) in multiple cancers. We first investigated the expression of *PLK4* in six HCC cell lines (Huh7, BEL‐7402, G2.215, MHCC‐97L, MHCC‐97H, and HepG2), and found that *PLK4* was highly expressed in Huh7 and BEL‐7402 (Figure 4A). We further divided the cells to groups of *PLK4*‐low and *PLK4*‐high, and used MTT assay to evaluate the inhibitory effect of CFI‐400945 on HCC cells. First, we found PLK4 were totally inhibited in Huh7 cell (Figure 4B). Furthermore, CFI‐400945 was found to inhibit cell proliferation at very low concentrations in a dose‐dependent manner. Cells with high‐*PLK4* expression showed more sensitivity to CFI‐400945 than cells with low‐*PLK4* expression (Figure 4C). In addition, this inhibition almost disappeared when *PLK4* expression was temporarily knocked down by siRNAs (Figure 4D). This indicated that by specifically inhibiting PLK4, CFI‐400945 could restrict the uncontrolled expansion of HCC cells.

## 4 DISCUSSION

In our association analysis, the CT gene *PLK4* was implicated as the HCC‐susceptibility gene tagged by a common variant rs3811741 in its enhancer region. Our *cis*‐eQTL analysis revealed that the risk allele of SNP rs3811741 is significantly associated with increased *PLK4* expression in HCC tissues in TCGA database. Finally, we validated the oncogenic effects of *PLK4* on HCC cells’ vitality in vitro.

PLK4 is a critical regulator of centriole duplication that has a crucial role in maintaining mitotic fidelity.^12^, ^15^ Plenty of evidences proved that overexpression of *PLK4* in somatic cells leads to centriole amplification.^21^, ^22^ Recently, studies found that centriole amplification was a direct causative factor for CIN and tumorigenesis.^23^, ^24^, ^25^, ^26^ In mammals, Levine* et al* revealed that supernumerary centrosomes induced by PLK4 were sufficient to drive aneuploidy and the development of spontaneous tumors in multiple tissues in mouse model.^27^, ^28^ In this study, we found the expression of *PLK4* in liver cancer tissues is much higher than that in adjacent normal liver tissue. Moreover, patients with high expression of *PLK4* had a poor prognosis.^29^, ^30^ These phenomena revealed that *PLK4* was abnormally activated and the activation was correlated with poor prognosis. To further validate the oncogenic effects of PLK4, we investigated the effects of PLK4 on cancer cell functions in HCC cell lines. Our data showed that by inducing *PLK4* silencing or overexpression, the biological characteristics of HCC cells could be significantly changed in the dimensions of proliferation, migration and invasion, being weakened after silencing, and being strengthened after overexpression.

However, previous studies have suggested that downregulated *PLK4* expression, mainly due to LOH,^31^, ^32^ is likely a driving factor for HCC formation. The incidence of spontaneous HCC in *Plk4* ^+/−^ mice was about 15 times higher than that of *Plk4* ^+/+^ mice.^32^ In contrast, other studies reported that *PLK4* overexpression results in centriole amplification,^21^, ^33^ while its depletion prevents centriole duplication.^33^ Both of the process caused loss of centrosome numeral integrity that led to aneuploidy and triggering tumorigenesis.^16^, ^34^ Thus, we speculate that PLK4 is a dose‐dependent risk factor and causes aneuploidy to promote HCC.

As stated before, being a serine threonine kinase, PLK4 phosphorylates a set of substrates such as Cep135, to correctly position the essential centriole component Asterless and PCM1, to maintain centriolar satellite integrity.^35^, ^36^ Meanwhile, PLK4 can also be ubiquitinated as a result of auto‐phosphorylation, thus help its self‐destruction and preventing centrosome amplification.^37^ However, the decompensated overexpression of *PLK4* directly results in centrosome amplification and thus leading to aneuploidy. As previous studies have demonstrated, the overexpression of *PLK4* causes aneuploidy in p53‐deficient progenitors and promotes the tumorigenesis of skin epidermis through transient centriole amplification.^38^ Moreover, PLK4 also contributes to the invasion and metastasis of many types of cancer. The downregulation of *PLK4* is able to inhibit the process of epithelial‐mesenchymal transition (EMT) both in vivo and in vitro, which is related to cancer metastasis.^39^ Therefore, the overexpression of *PLK4* in HCC that we have found reveals its oncogenic role in promoting the generation of aneuploidy and cancer metastasis. However, we have not demonstrated the specific mechanism in this paper and it needs further verification.

*PLK4* presents CT gene expression pattern due to its limited expression in normal tissues and wide distribution in tumors. Besides, recent studies have found that CT gene also harbors a cancer‐promoting effect.^10^, ^40^ Therefore, accumulating articles believe oncogenic role of CT gene could be an ideal window of targeted therapy for tumors.^41^ For example, LIN28B, a newly defined CT gene, participated in the LIN28B/let‐7 axis that could be used to target *MYC* in multiple myeloma or other *MYC*‐dependent cancers.^42^ Besides, therapies targeting the classic CT gene MAGE‐A have already been put into clinical trail.^43^, ^44^ At present, the treatment of HCC, especially advanced HCC, faces great challenges. HCC is not sensitive to chemotherapy, and targeted drugs such as sorafenib and regorafenib have certain benefits, but far below clinical expectations.^45^, ^46^ Since *PLK4* is abnormally activated and plays an oncogenic role in part of HCC, it is likely to be a therapeutic target for HCC. Therefore, we conducted DST of CFI‐400945, a small molecule inhibitor of PLK4 undergoing phase I/II clinical trial testing (NCT03187288; NCT01954316; NCT03624543; NCT03385655). Based on the loss of centrosome numeric integrity, the inhibition of PLK4 by CFI‐400945 would further disrupt centriole duplication, thus triggering the death of cancer cells.^47^ Our results have also demonstrated that CFI‐400945 suppressed HCC proliferation by specifically targeting PLK4. The inhibitory potency was dose‐dependent and was stronger in cell lines with high background expression in contrast to *PLK4*‐null cell lines. The inhibition efficiency was significantly weakened or nearly disappeared after *PLK4* was silenced. These results provide an experimental basis for PLK4 to become a potential therapeutic target for HCC.

In short, our results suggest that the *cis*‐eQTL genetic variant in *PLK4* confers risk to HCC. Furthermore, *PLK4* sensitives the HCC to CFI‐400945, a small molecule inhibitor of PLK4 undergoing phase I/II clinical trial testing. Subsequent studies are needed to explore anti‐PLK4 therapy in animal studies and even clinical trials in HCC.

## CONFLICT OF INTEREST

None declared.

## AUTHOR CONTRIBUTIONS

ZH, GY, RL and XH initiated, conceived and supervised the study. LM performed the population studies and data analysis with JH, WY, CS, CW, and JD; LM, YZ, YG, SJ, JK, YW, SL, and JX conducted the in vitro experiments; LM, drafted the manuscript with YG YZ, GJ, HM, and HS.
