# Supplementary figures and images for: A cis‐eQTL genetic variant in PLK4 confers high risk of hepatocellular carcinoma
Source: Cancer Med. 2019 Sep 6;8(14):6476–84. doi: 10.1002/cam4.2487 (PMC6797585; doi:10.1002/cam4.2487)

A

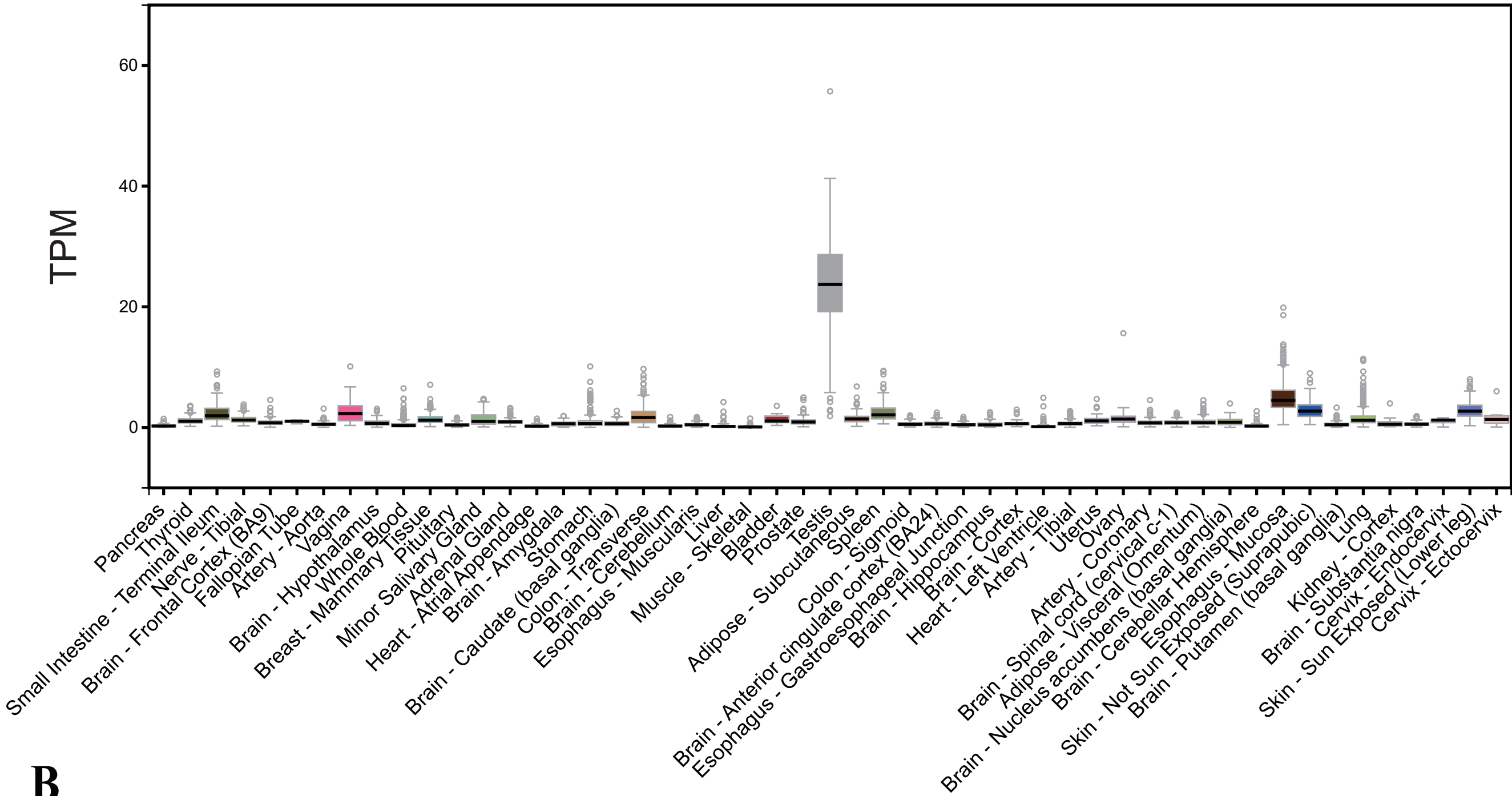

B

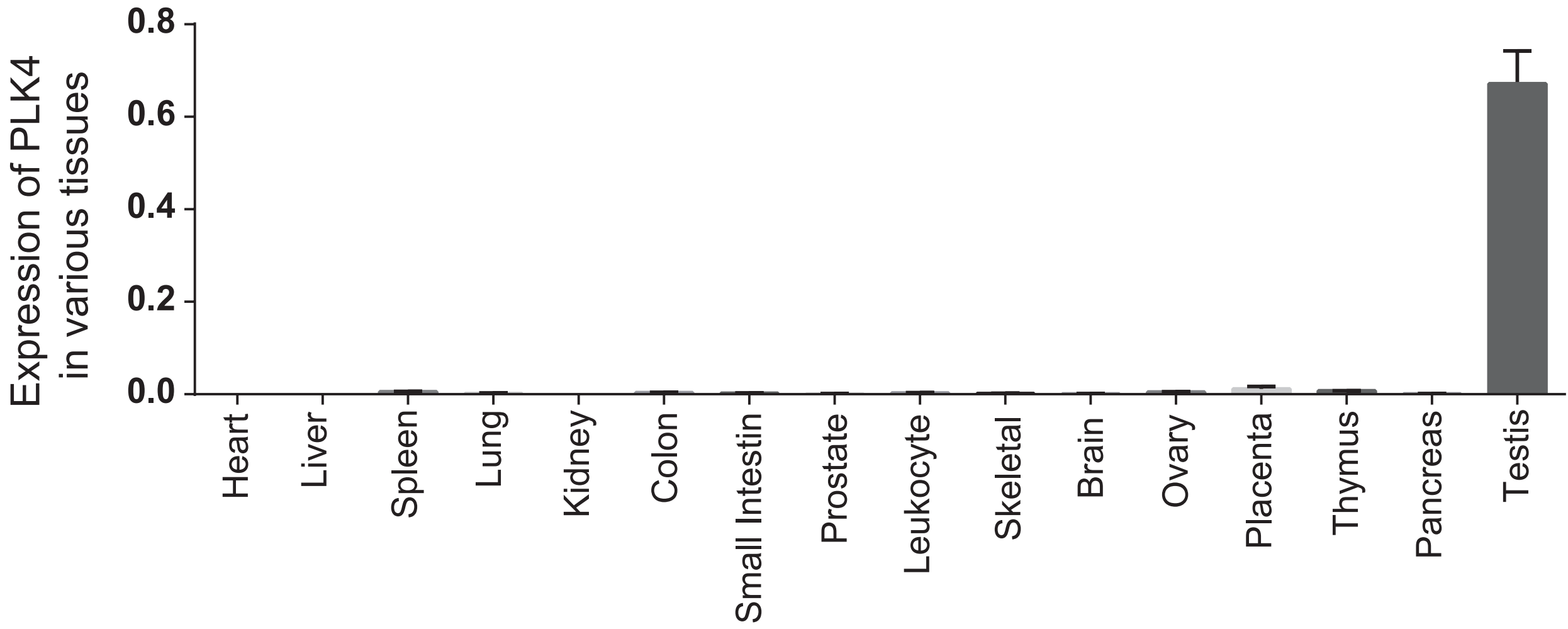

Supplement: Supplementary file 1 [file CAM4-8-6476-s001.pdf]

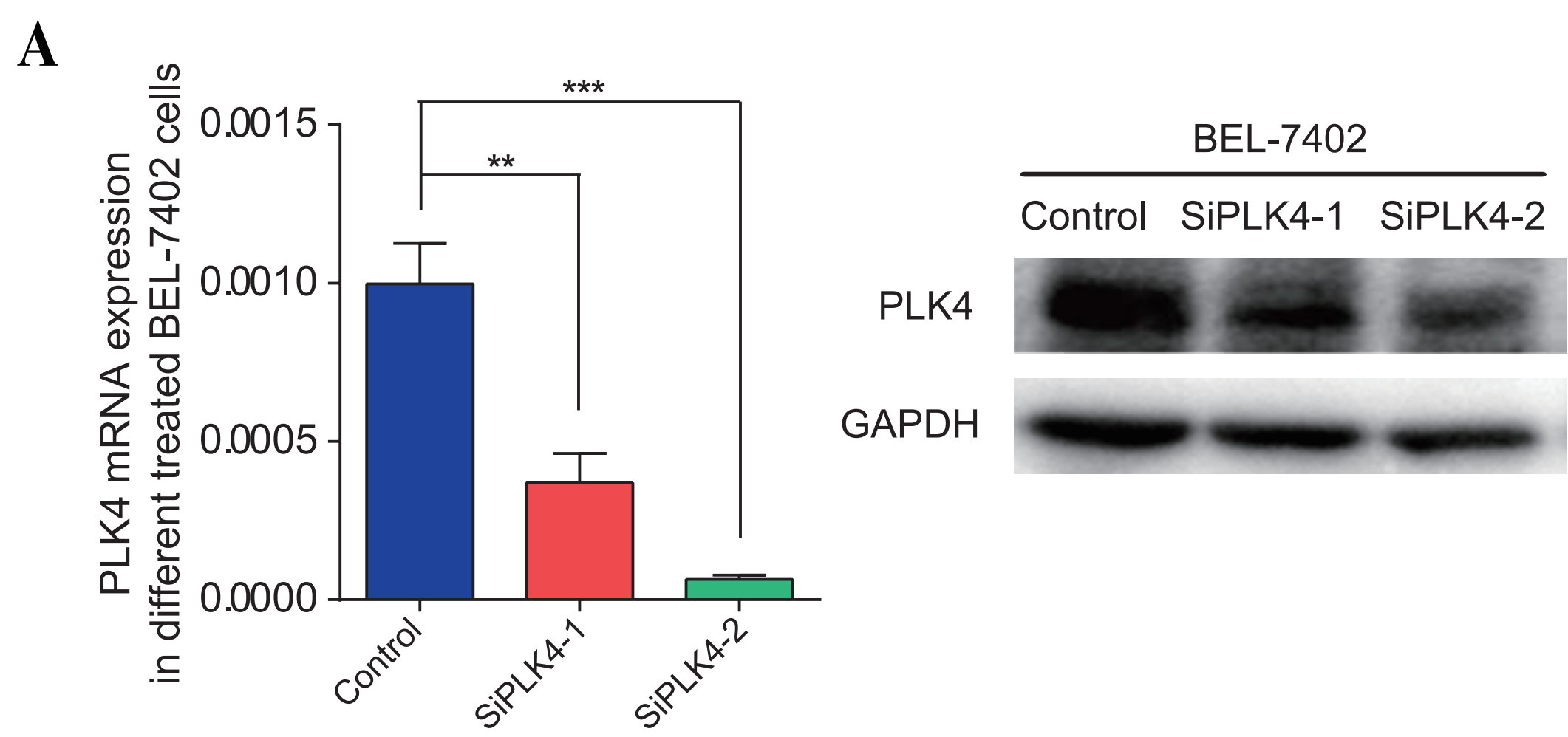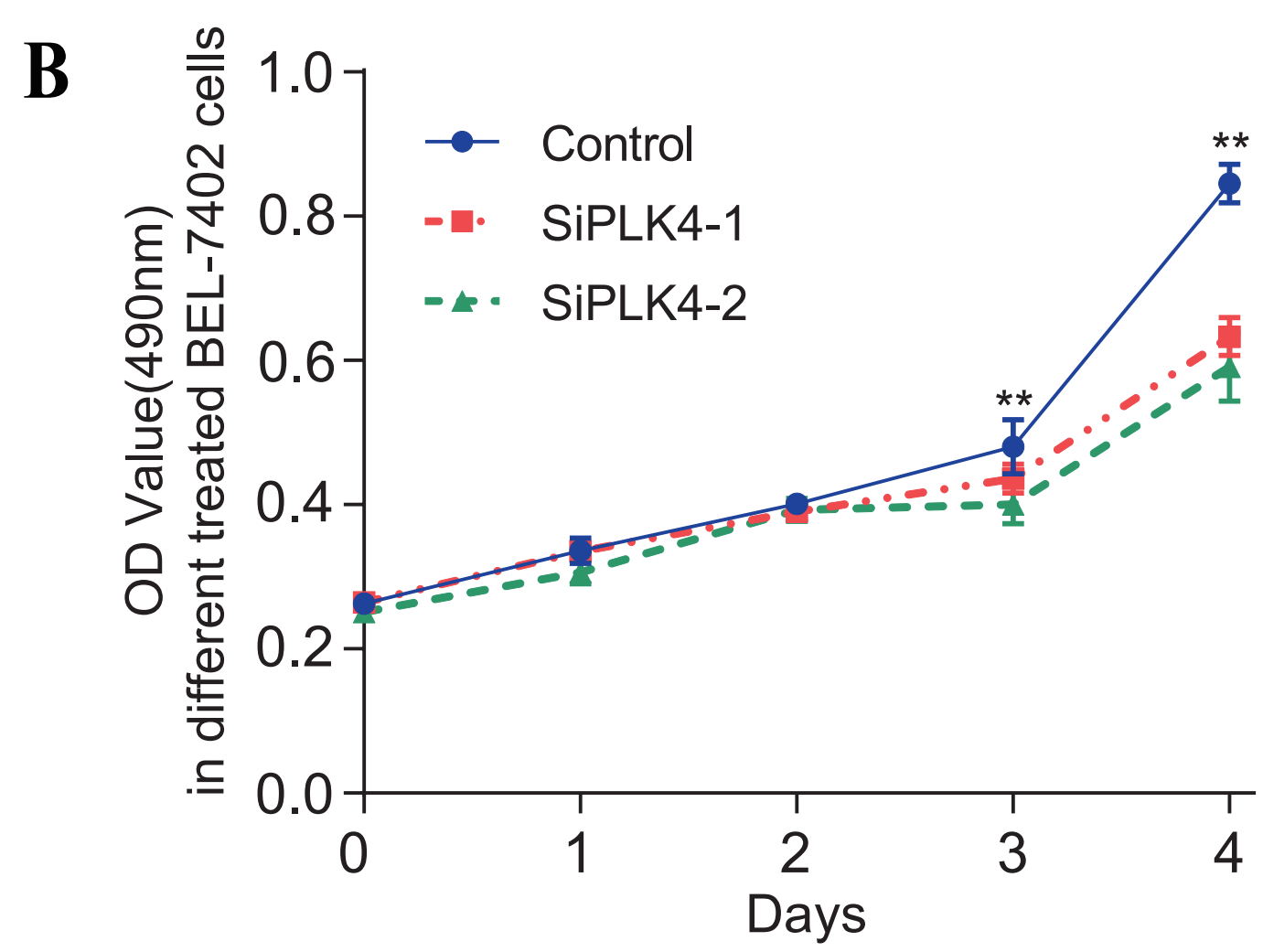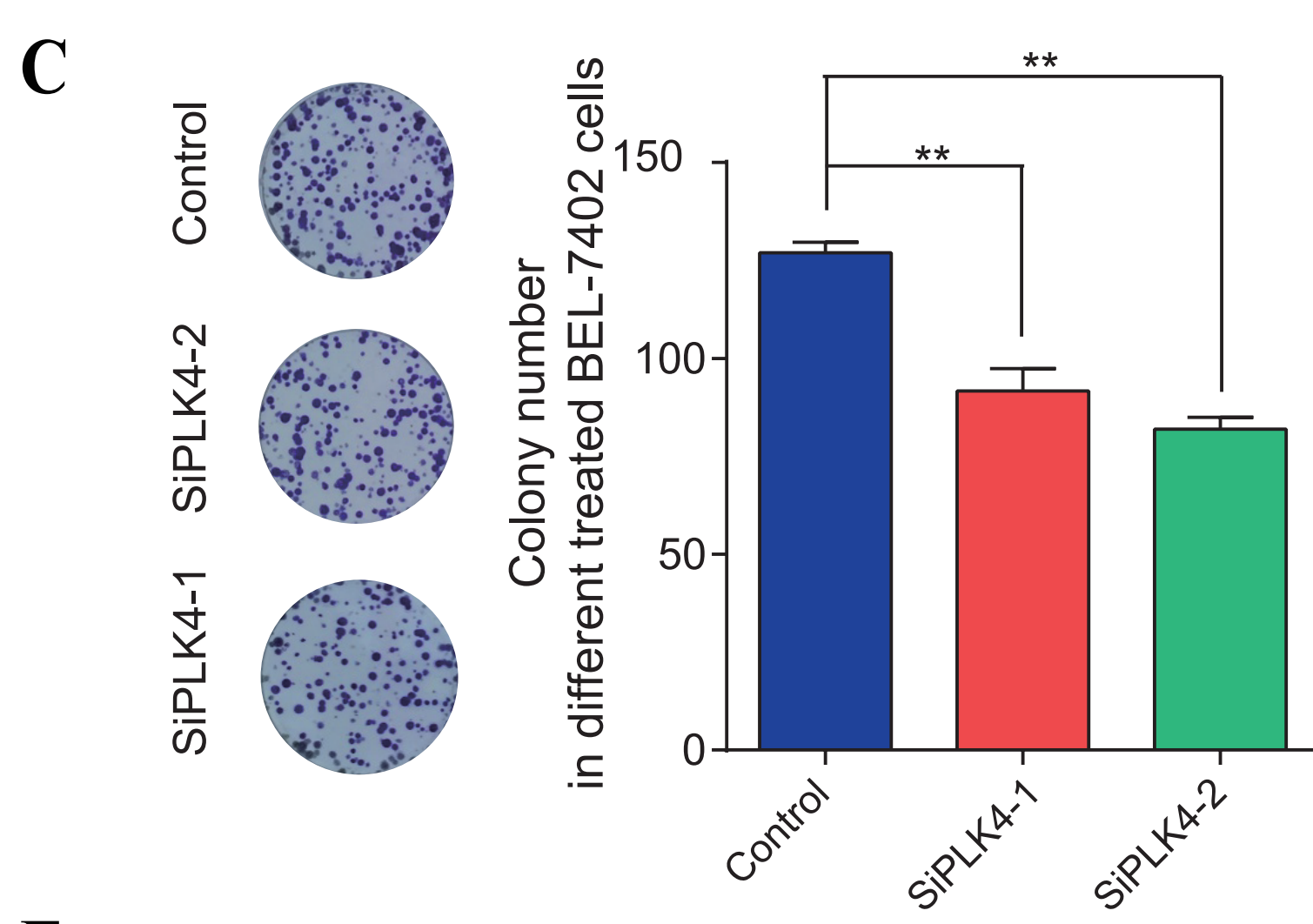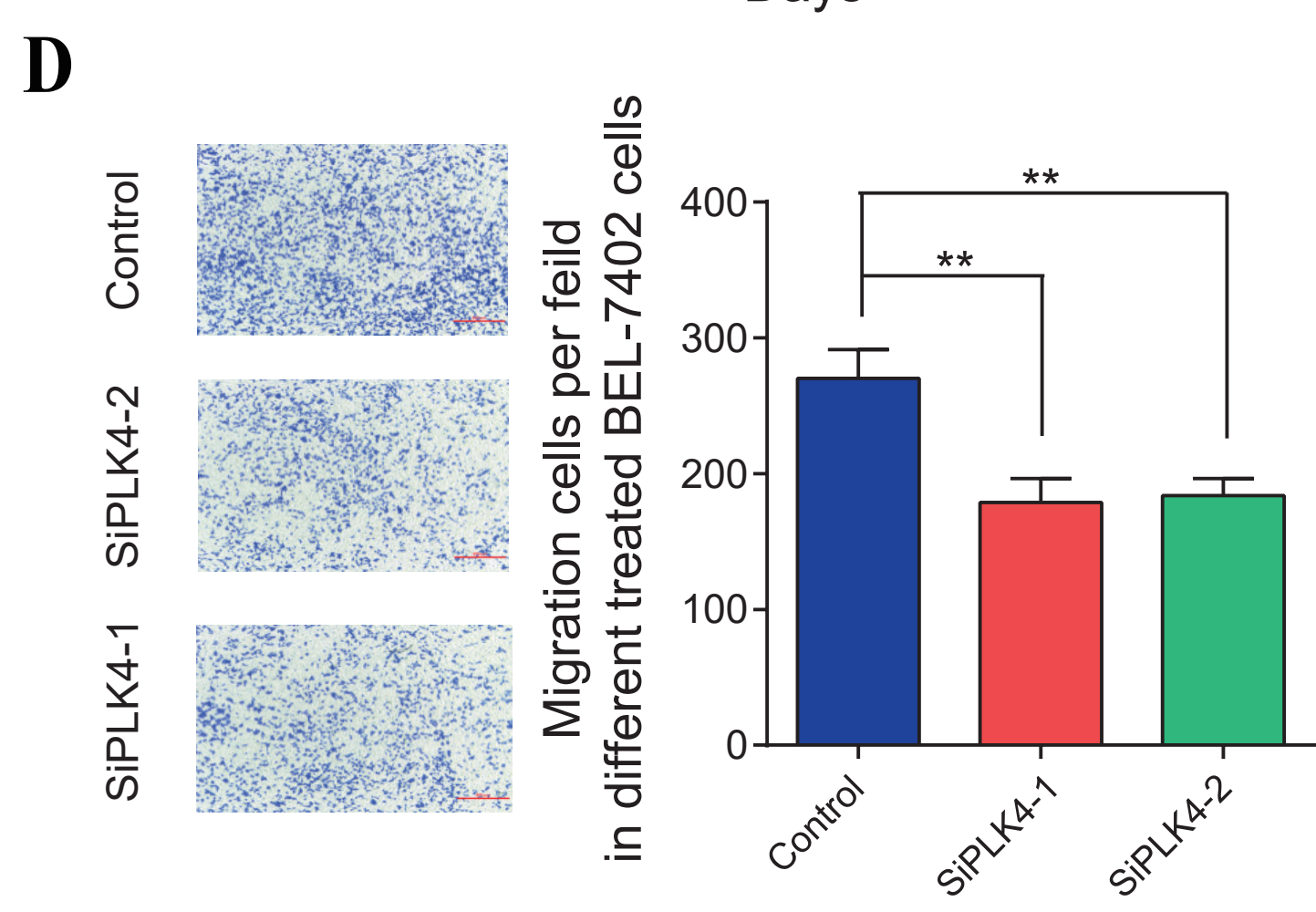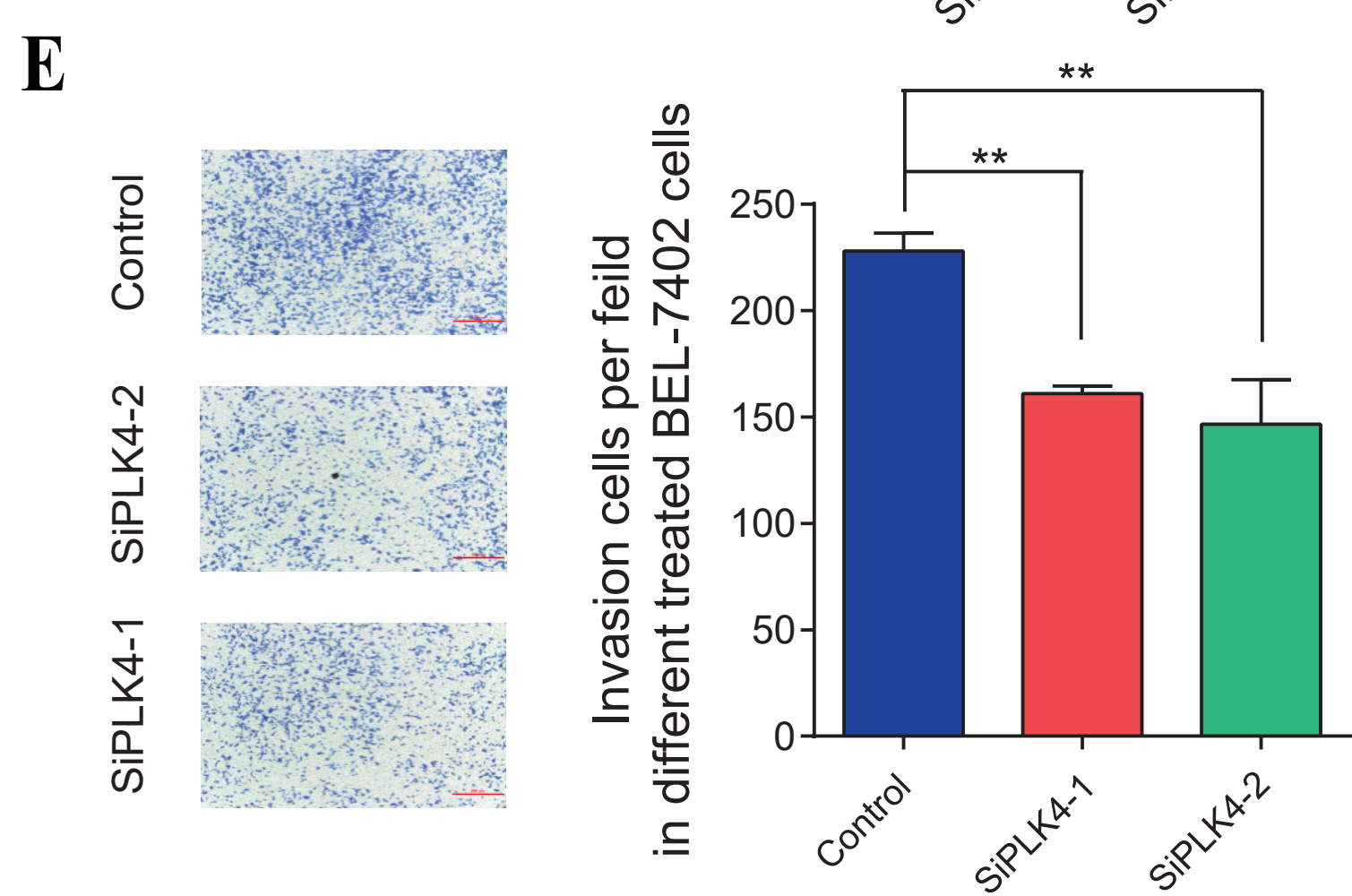

Supplement: Supplementary file 2 [file CAM4-8-6476-s002.pdf]

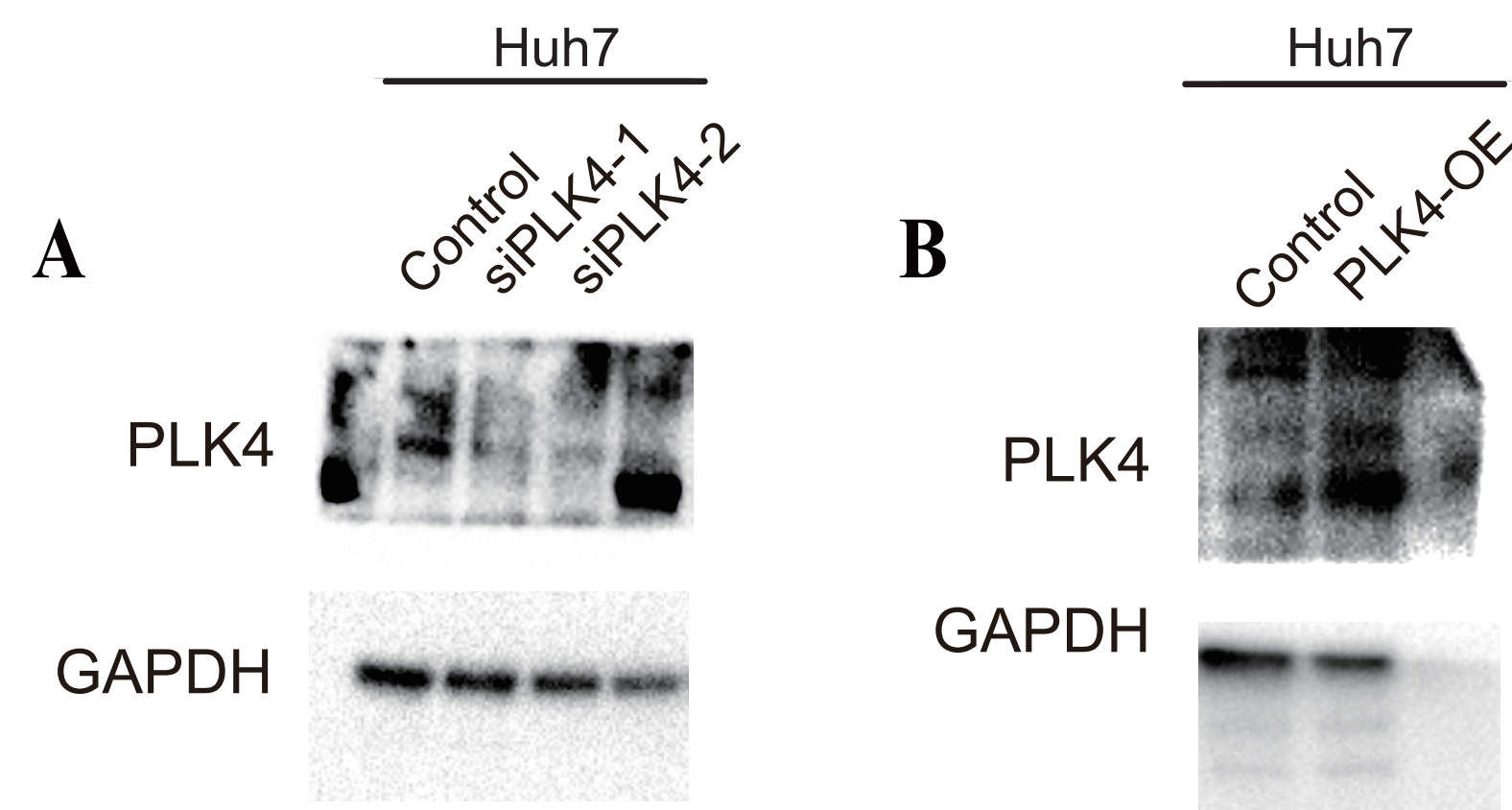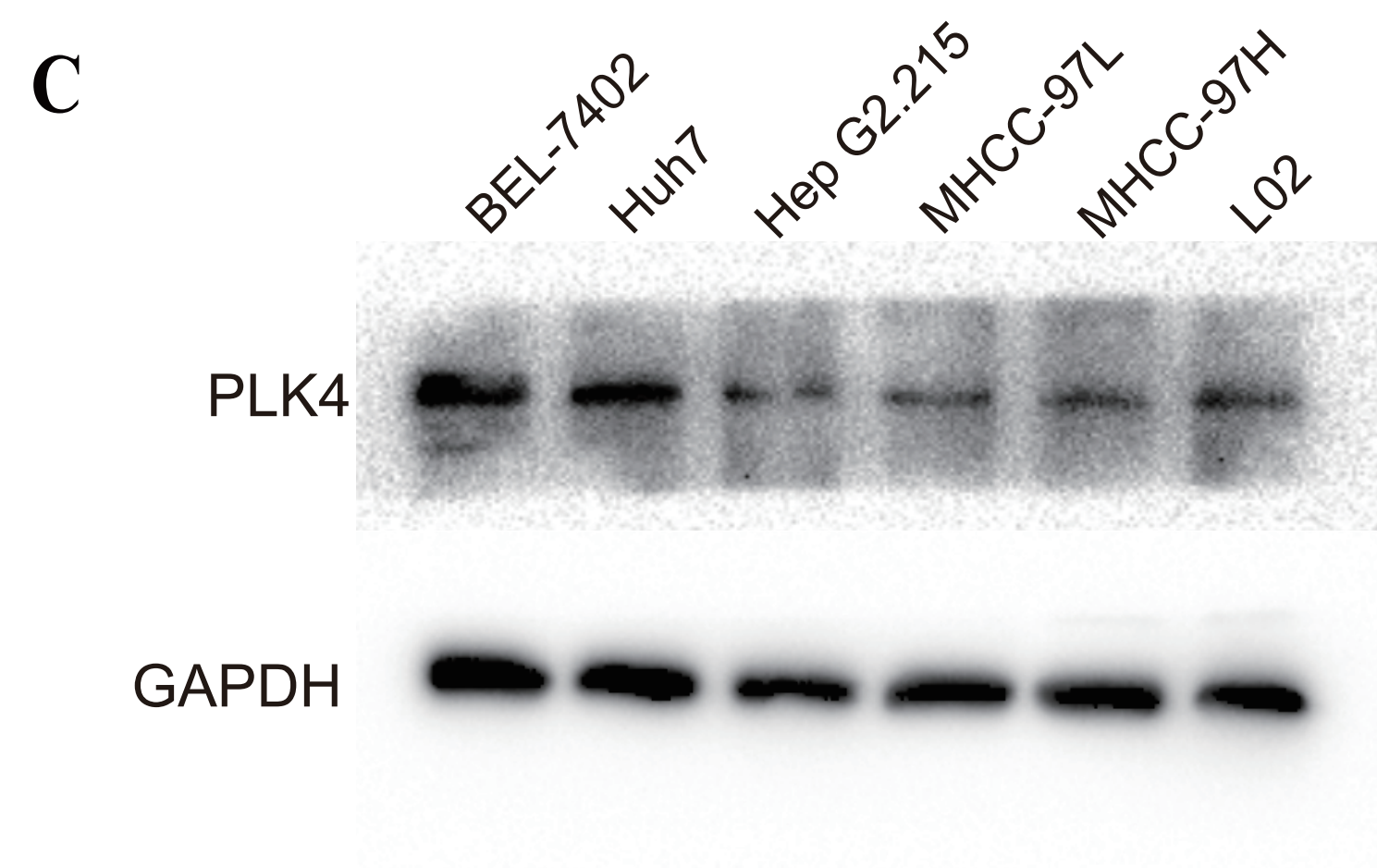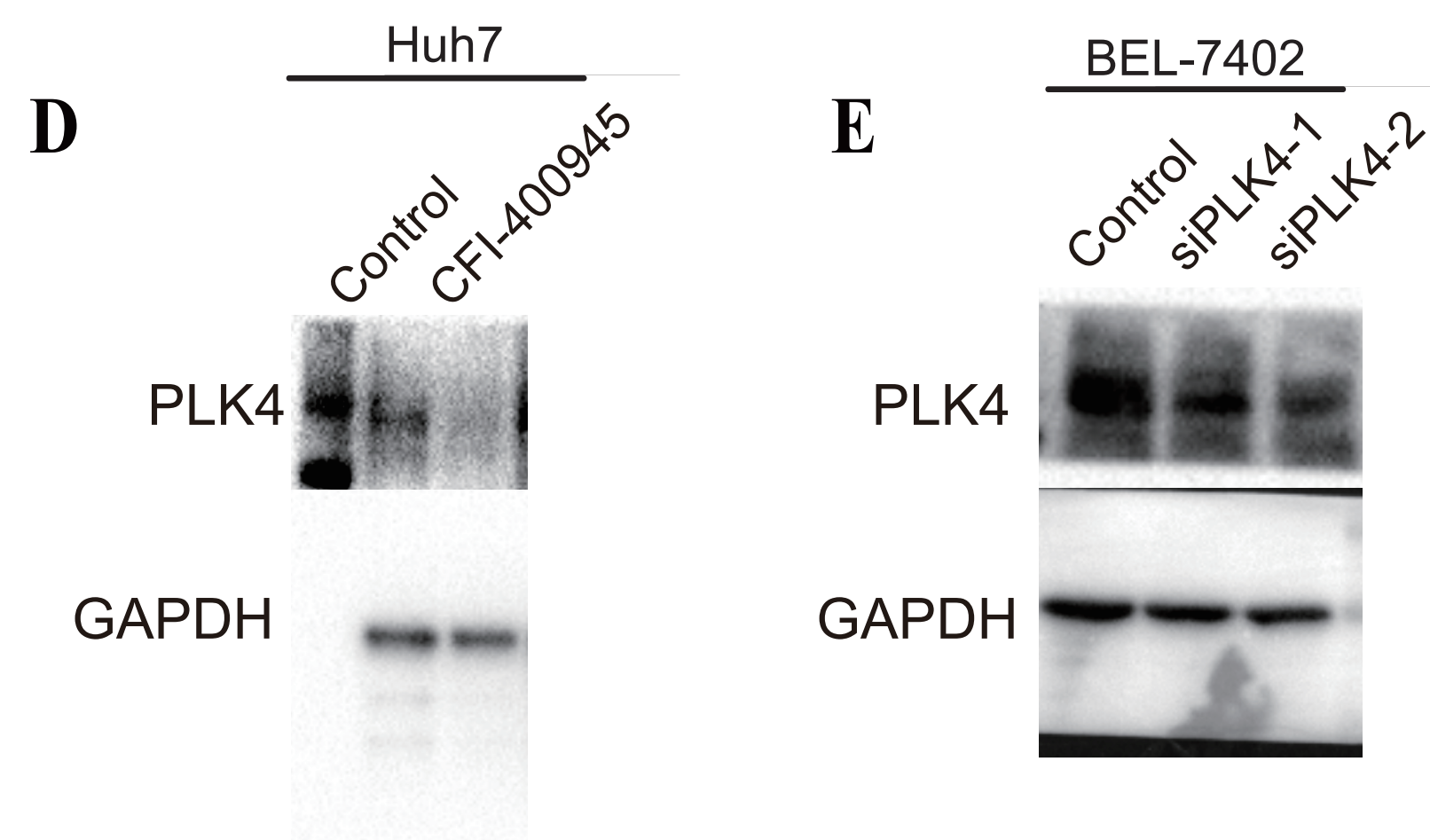

Supplement: Supplementary file 3 [file CAM4-8-6476-s003.pdf]
